# Brain health in association football: perspectives of professional players

**DOI:** 10.3389/fspor.2026.1876247

**Published:** 2026-08-18

**Authors:** J. Batten, A. J. Pearce, N. E. Howarth, H. Dawes, D. H. Daneshvar, C. J. Nowinski, A. J. White

**Affiliations:** 1 Department of Sport, Allied Health Professions and Social Work, Faculty of Health and Wellbeing, University of Winchester, Winchester, United Kingdom; 2 Swinburne Neuroimaging Facility, School of Health Sciences, Swinburne University, Melbourne, VIC, Australia; 3 NIHR Exeter Biomedical Research Centre, University of Exeter Medical School, St Luke’s Campus, Exeter, United Kingdom; 4 Warwick Medical School, University of Warwick, Coventry, United Kingdom; 5 Department of Physical Medicine and Rehabilitation, Harvard Medical School, Boston, MA, United States; 6 The Concussion and CTE Foundation, Boston, MA, United States; 7 The Professional Footballers’ Association, Manchester, United Kingdom

**Keywords:** brain health, concussion, concussion education, concussion reporting, player welfare, professional football, retired athletes, sport-related concussion

## Abstract

**Introduction:**

Growing scientific and public concern regarding concussion and the potential long-term neurological consequences of playing professional football has increased attention on brain health within the sport. However, relatively little research has examined professional players’ perspectives on concussion management and brain health risks.

**Method:**

A cross-sectional, anonymous, online survey was distributed to members of the Professional Footballers’ Association (PFA) in March 2023. The survey assessed concussion history, attitudes towards concussion management, support for temporary concussion substitutions, perceptions of heading-related risks, concerns about long-term neurological conditions, as well as engagement with concussion education. Responses from 600 current and former professional football players (97% male; 3% female) across multiple playing levels were analysed using descriptive statistics and non-parametric tests.

**Results:**

A larger proportion of players reported having suspected a concussion during their career than reported receiving a formal diagnosis. Although many players indicated a preference to remain on the field following a suspected concussion, most believed that athletes should not be responsible for deciding whether to continue playing. Strong support was observed for the introduction of temporary concussion substitutions, particularly among retired players and those with longer playing careers. Many respondents also expressed concern regarding heading-related risks and the potential development of long-term neurological conditions. Players who had received concussion education demonstrated greater awareness of these risks.

**Discussion:**

Professional football players acknowledged important challenges in concussion recognition and management and broadly support policy measures aimed at improving player safety. The findings highlight potential gaps in concussion diagnosis and education within the professional game and suggest that players favour approaches that include thorough medical assessment and reduce pressures to continue playing following head injury. Incorporating athlete perspectives into policy development may help inform effective concussion management strategies and support efforts to protect the long-term brain health of professional footballers.

## Introduction

A concussion is defined as a traumatic brain injury induced by biomechanical forces that produces a range of clinical symptoms, including headache, dizziness, cognitive impairment, and disturbances in balance or sleep (1). In association football (soccer) - henceforth football - epidemiological studies estimate concussion incidence rates from between approximately 0.5 and 1.7 injuries per 1,000 match hours (2, 3). More recently, research (4) has found that concussions accounted for 7% of all injuries in women's international football, 5% in women's domestic, 3% in men's international, and 2% in men's domestic. While current international consensus guidelines recommend immediate removal from play following suspected concussion, followed by a graduated return-to-play protocol once symptoms have resolved (1), concussion recognition often depends on players self-identifying and reporting symptoms to medical staff. However, research across multiple sports suggests athletes may underreport symptoms due to competitive pressures, uncertainty about injury severity, or cultural norms that prioritise toughness and resilience (5). Consequently, athletes play a critical role in current approaches to concussion recognition and management, making their knowledge, attitudes, and perceptions of brain injury particularly important.

To facilitate the assessment of suspected concussion during matches, football has head injury assessment protocols that allow medical staff to evaluate players following head impacts. However, these assessments often occur under time constraints and may require teams to play with fewer players temporarily, which can create competitive disadvantages (6). Although additional permanent concussion substitutes allow players with a suspected concussion to be removed from a match without counting towards a team's allocated substitutions, the implementation of such substitutes in the Premier League in the 2020–2021 season did not impact the rate or duration of medical assessment of head collision events (7). As a result, there has been increasing discussion regarding the potential implementation of temporary concussion substitutions in football (8). Such substitutions would allow a player to leave the field for a comprehensive medical assessment without disadvantaging their team. Temporary concussion substitutions have been implemented in several other sports, including rugby union, where they have been shown to support more thorough medical evaluations and improve player safety (9). While the potential adoption of similar measures has been debated by the International Football Association Board - which is responsible for determining the laws of the game - as yet, views from professional football players themselves have not been sought on this issue.

Beyond the acute management of concussion, there is increasing concern regarding the potential long-term neurological consequences of repeated head impacts in sport that may cause neurodegenerative conditions like Chronic Traumatic Encephalopathy (CTE) (10, 11). In football, attention has focused on the cumulative effects of heading the ball and other repetitive head impacts sustained throughout a player's career. Here, epidemiological research has suggested that former professional footballers may have an increased risk of neurodegenerative diseases compared with the general population. For example, the FIELD Study reported that former professional football players had a higher incidence of neurodegenerative disease mortality relative to matched population controls (12). Further, a recent meta-analysis has demonstrated greater rates of neurodegenerative disease in footballers compared to the general population (13). Neuropathological investigations in former athletes from several contact sports have also identified CTE (14). These findings have increased public and scientific scrutiny of the brain health risks associated with participation in sport (15). Again, however, the views of professional football players are often missing from these discussions.

Alongside epidemiological and neuropathological research, education programmes have been widely promoted for improving concussion recognition and management among athletes (16). Typically, concussion education programmes aim to enhance players’ knowledge of symptoms, reinforce the importance of reporting head injuries, as well as encourage adherence to return-to-play guidelines. Here, previous research [e.g., (17)] suggests that education programmes can improve athletes’ concussion knowledge and attitudes. However, the relationship between knowledge and behaviour remains complex, with improvements in awareness not always translating into increased symptom reporting or safer decision-making during competition (16). In addition to influencing injury reporting behaviours, concussion education may also shape athletes’ perceptions of brain health risks, including their views on heading exposure, long-term neurological consequences, as well as rule changes designed to improve player safety.

Despite the central role that athletes play in recognising and reporting concussion symptoms, their perspectives have often been underrepresented in research and policy discussions related to concussion in sport (15). Indeed, decisions regarding rule changes, safety protocols, and medical procedures are largely driven by governing bodies, clinicians, and researchers, despite players themselves being directly affected by these policies. Thus, incorporating athlete perspectives, as those directly affected, into discussions surrounding injury prevention and player welfare is essential, with players’ experiences and attitudes providing valuable insights into the practical realities of concussion recognition and reporting, as well as potential barriers to implementing concussion interventions (18). Yet, relatively little work has explored professional football players’ perceptions of brain health, concussion management strategies, and long-term neurological risk. Specifically, there is limited evidence regarding players’ views on temporary concussion substitutions, their concerns about heading-related risks, and the potential influence of concussion education and career exposure on these perceptions.

The present study addresses this gap by examining the perceptions of current and former professional football players regarding brain health. Using survey data collected from members of the Professional Footballers’ Association (PFA), this study explores players’ experiences of concussion, attitudes towards concussion management and temporary concussion substitutions, as well as their concerns regarding heading-related risks and potential long-term neurological outcomes such as CTE. Further, this study examines whether playing status, professional playing experience, and concussion education impact these perceptions. By capturing the perspectives of professional football players, this research aims to contribute to a more comprehensive understanding of brain health in football and to inform ongoing discussions regarding concussion management, player safety, as well as policy development within the sport.

## Method

### Design

This study used a cross-sectional survey to examine professional football players’ perspectives on brain health. A cross-sectional design supported the collection of standardised data from the identified population at a single point in time, allowing patterns in knowledge, attitudes, and experiences related to brain health to be identified across participants. The use of a survey was appropriate within this elite sport context, where time and access constraints limit more resource-intensive approaches (e.g., interviews). Further, the approach used in this study aligns with previous literature [e.g., (15)] that has called for sport concussion and CTE research to include athletes’ perspectives and lived experiences. Indeed, concussion and CTE research is often dominated by clinical and biomedical perspectives, overlooking insights from players who are directly exposed to head impacts and whose behaviours and decision-making influence injury reporting and management. Thus, examining players’ perspectives supports an athlete-centred approach to understanding brain health in sport and can help inform future education, policy, and prevention initiatives.

### Participants

Participants were recruited through the PFA mailing list as part of routine communications sent to members. All current PFA members were eligible to participate, including both current and former professional football players. Participation was voluntary, with participants informed that responses would remain confidential and anonymised, that no personally identifiable information would be reported, that they could withdraw from the study at any point prior to submission of the survey without consequence, and that data provided would be used to inform research and policy. To be included in the analysis, participants were required to be PFA members and complete all survey questions (incomplete responses were excluded). No other exclusion criteria were applied.

A total of 600 members responded to the survey. The sample comprised 582 men (97%) and 18 women (3%). Male respondents represented multiple levels of the English football pyramid, including the Premier League, Championship, League One, and League Two, while female respondents competed in the Women's Super League and FA Women's Championship. Retired players made up the largest group of respondents (67.3%), including both male and female former professional football players. Participants also reported the duration of their playing careers, with over half of respondents (56.7%) indicating they had competed for 11 or more seasons. Biological sex is reported to contextualise analyses. However, the relatively small number of female participants limited sex-specific subgroup analyses (see Tables 1, 2).

**Table 1:** Playing status of survey respondents.

Playing Status	Number of Responses	Proportion of Sample (%)
Premier League	6	1.0
Championship	40	6.7
League One	31	5.2
League Two	101	16.8
Women's Super League	15	2.5
FA Women's Championship	3	0.5
Retired^a^	404	67.3

a Retired players include both male and female respondents from across multiple leagues.

**Table 2:** Years of professional playing experience.

Years Played	Number of Responses	Proportion of Sample (%)
1–3 Years	69	11.5
4–6 Years	101	16.8
7–10 Years	90	15.0
11 + years	340	56.7

### Measures

Data were collected using an online survey hosted on SurveyMonkey. The survey was available for a two-week period in March 2023 and was distributed to members through regular email communications from the PFA. The communication sent to members included a brief description of the study and a link to the survey. The survey was internally reviewed (and approved) by the PFA prior to distribution. Responses were collected and stored on secure PFA servers and anonymised prior to transfer for secondary analysis in accordance with relevant data protection procedures. This process was approved by the institutional ethics committee at the University of Exeter.

To contextualise responses, the survey started with questions on playing history and status. Specifically, playing experience was assessed with the question: “*How many years have you played football full-time (including scholarship)?”* with responses ranging from between 1 and 3 years to 11 years or more. At the same time, current playing status was assessed with the item: “*What is your current playing status?”* (retired, Premier League, Championship, League One, League Two, National League or below, Women's Super League, FA Women's Championship or below).

Next, participants reported their personal experiences with concussion through the following items: “*Have you ever experienced a suspected concussion while playing football?”* (yes, no) and “*Have you ever been diagnosed with a concussion from playing football?”* (yes, no). Attitudes towards in-game concussion management were assessed with the question: “*If you were concussed during a game, would you want to be substituted or stay on and continue playing?”* [always stay on, stay on (if possible), unsure, always come off]. Participants were also asked: “*If you are concussed, who should make the decision whether you stay on the field?”* (player, other).

Considering concussion management and brain health in football, participants were asked: “*To what extent do you support the call to trial temporary concussion substitutions in football?”* with responses recorded on a five-point Likert scale (strongly favour, somewhat favour, neutral, somewhat oppose, strongly oppose).

Concern about the potential risks associated with heading a football was assessed using the question: “*To what extent are you concerned about the risks associated with heading a football?”* (extremely concerned, moderately concerned, somewhat concerned, slightly concerned, not at all concerned). Participants were also asked about concerns regarding longer-term neurological risks through the item: “*To what extent are you concerned about your long-term brain health, and the potential risks of developing dementia or chronic traumatic encephalopathy (CTE)?”* (extremely concerned, moderately concerned, somewhat concerned, slightly concerned, not at all concerned).

Finally, participants were asked about their engagement with concussion education programmes. Specifically, participants were asked: “*Have you ever received education about concussion?”* (yes, no).

### Data analysis

Data were analysed using the Statistical Package for Social Sciences (SPSS v.31). First, descriptive statistics were calculated to summarise participant characteristics and survey responses. Here, frequencies and percentages were reported for concussion experience (diagnosed and suspected concussions, preferences for in-game concussion management, removal from play decisions), support for temporary concussion substitutions, concerns about heading the ball and long-term brain health and CTE risk, as well as concussion education programme completion. Second, the distribution of data was assessed using Kolmogorov–Smirnov tests prior to inferential statistical analyses. These analyses indicated that data were not normally distributed (*p* < 0.05). Consequently, non-parametric statistical tests were employed throughout. To examine for differences in survey responses according to playing status and years of professional playing experience, Kruskal–Wallis tests for differences were conducted. Effect size was calculated using Epsilon-Squared (*ε*^2^), with results interpreted using values < 0.01 as negligible, from between 0.01–0.08 as small, from between 0.08–0.26 as medium, and values ≥0.26 as large. Following a significant (*p* < 0.05) Kruskal–Wallis test result, *post-hoc* analyses using Dunn's tests with Bonferroni corrections were undertaken. Differences in survey responses between those who had and those who had not received concussion education were examined using Mann–Whitney U tests, with statistical significance set at *p* < 0.05. Here, effect sizes were calculated using the rank-biserial correlation, with r values ≥ 0.10 interpreted as small, ≥0.30 as medium, and ≥0.50 as large.

## Results

### Concussion experience

When asked about their concussion history, slightly more respondents reported that they had not been formally diagnosed with a concussion while playing football compared with those who had (*n* = 326 “no”, *n* = 274 “yes”). In contrast, most players indicated that they had experienced a suspected concussion while playing football (*n* = 435 “yes”, *n* = 165 “no”).

Regarding preferences for in-game concussion management, the most common response (*n* = 297) to the question “*If you were concussed during a game, would you want to be substituted or stay on and continue playing?”* was “stay on, if possible” (49.5%).

Considering playing status, responses indicated significant differences in preferences for in-game concussion management [H _(6)_ = 32.364, *p* < 0.001], although the effect size was small (*ε*^2^ = 0.054). Here, 51% of retired players selected “stay on, if possible”, compared with 24.8% who indicated they would “always come off”. Among current players, responses varied by playing status. Premier League respondents were evenly split between “stay on, if possible” and “always come off”. Most Championship players selected “stay on, if possible” (57.5%), with 25% indicating “always come off”. In League One, the most frequent response was “always come off” (48.4%), followed by “stay on, if possible” (35.5%). In League Two, 49.5% selected “stay on, if possible”, while 34.7% indicated they would “always come off”. Respondents competing in women's leagues (Women's Super League, Women's Championship) most frequently selected “always come off” (66.7% in both groups) (see Table 3). *Post-hoc* analyses revealed significant differences in preferences for in-game concussion management between retired players and League One players (*p* = 0.022) and between retired players and Women's Super League players (*p* = 0.010) only.

**Table 3:** The impact of playing status on in-game concussion management decisions.

Playing Status	Response Option	Number of Responses	Proportion of Sample (%)
Premier League	Always Stay On	-	-
	Stay On, If Possible	3	50.0
	Unsure	-	-
	Always Come Off	3	50.0
Championship	Always Stay On	1	2.5
	Stay On, If Possible	23	57.5
	Unsure	6	15.0
	Always Come Off	10	25.0
League One	Always Stay On	1	3.2
	Stay On, If Possible	11	35.5
	Unsure	4	12.9
	Always Come Off	15	48.4
League Two	Always Stay On	4	4.0
	Stay On, If Possible	50	49.5
	Unsure	12	11.9
	Always Come Off	35	34.7
Women's Super League	Always Stay On	-	-
	Stay On, If Possible	4	26.7
	Unsure	1	6.7
	Always Come Off	10	66.7
FA Women's Championship	Always Stay On	-	-
	Stay On, If Possible	-	-
	Unsure	1	33.3
	Always Come Off	2	66.7
Retired	Always Stay On	60	14.9
	Stay On, If Possible	206	51.0
	Unsure	38	9.4
	Always Come Off	100	24.8

There were also significant differences in preferences for in-game concussion management by years of professional playing experience [H_(3)_ = 12.389, *p* = 0.006], with a small effect size (*ε*^2^ = 0.021). Specifically, players with shorter careers (1–3 years) showed a relatively higher proportion selecting “always come off” (39.1%) compared to other groups, suggesting a more cautious approach among less experienced players. In contrast, those with longer playing careers (11 + years) were more likely to report “always stay on” (13.8%). Nevertheless, across all career duration groups, the most common response was “stay on, if possible,” indicating a general preference among players to continue playing after a suspected concussion, when feasible. This pattern was consistent across all categories, ranging from 43.3% in those with 7–10 years of professional playing experience to 55.4% in those with 4–6 years (see Table 4). *Post-hoc* analyses indicated significant differences in preferences for in-game concussion management between those with 11 or more years of professional playing experience and those with from between 1 and 3 years of professional playing experience (*p* = 0.048), as well as between those with 11 or more years of professional playing experience and those with from between 7 and 10 years of professional playing experience (*p* = 0.048).

**Table 4:** The impact of years of professional playing experience on in-game concussion management decisions.

Years Played	Response Option	Number of Responses	Proportion of Sample (%)
1–3 Years	Always Stay On	3	4.3
	Stay On, If Possible	32	46.4
	Unsure	7	10.1
	Always Come Off	27	39.1
4–6 Years	Always Stay On	10	9.9
	Stay On, If Possible	56	55.4
	Unsure	9	8.9
	Always Come Off	26	25.7
7–10 Years	Always Stay On	6	6.7
	Stay On, If Possible	39	43.3
	Unsure	12	13.3
	Always Come Off	33	36.7
11 + years	Always Stay On	47	13.8
	Stay On, If Possible	170	50.0
	Unsure	34	10.0
	Always Come Off	89	26.2

When asked who should make the decision about removal from play following a suspected concussion, most players (*n* = 477) reported that athletes should not be responsible for determining whether it is safe to continue playing (79.5%).

Here, no significant differences were observed according to playing status [H _(6)_ = 9.343, *p* = 0.155, *ε*^2^ = 0.016], with the proportion of players reporting that athletes should not be responsible for making decisions about removal from play ranging from between 53.3% in the Women's Super League to 81.2% among retired players. All Premier League (*n* = 6) respondents reported that players should not be responsible for determining whether it is safe to continue playing (see Table 5).

**Table 5:** The impact of playing status on removal from play decision-making.

Playing Status	Response Option	Number of Responses	Proportion of Sample (%)
Premier League	Player	-	-
	Other	6	100.0
Championship	Player	9	22.5
	Other	31	77.5
League One	Player	7	22.6
	Other	31	77.4
League Two	Player	23	22.8
	Other	78	77.2
Women's Super League	Player	7	46.7
	Other	8	53.3
FA Women's Championship	Player	1	33.3
	Other	2	66.7
Retired	Player	76	18.8
	Other	328	81.2

Similarly, comparison of years of professional playing experience revealed no significant differences in preferences for removal from play decision-making [H _(3)_ = 2.360, *p* = 0.501], with a negligible effect size (*ε*^2^ = 0.004). Specifically, across all career duration groups, most players indicated that the decision to be removed from play following a suspected concussion should be made by someone other than the player themselves. This pattern was consistent across all categories, with responses ranging from between 74.4% (among those with 7–10 years of professional playing experience) to 83.2% (among those with 4–6 years of professional playing experience) (see Table 6).

**Table 6:** The impact of years of professional playing experience on removal from play decision-making.

Years Played	Response Option	Number of Responses	Proportion of Sample (%)
1–3 Years	Player	13	18.8
	Other	56	81.2
4–6 Years	Player	17	16.8
	Other	84	83.2
7–10 Years	Player	23	25.6
	Other	67	74.4
11 + years	Player	70	20.6
	Other	270	79.4

### Support for temporary concussion substitutions

Overall support for the introduction of temporary concussion substitutions was high. Most respondents selected “strongly favour” (*n* = 352), followed by “somewhat favour” (*n* = 116). In contrast, 8.5% of respondents indicated some level of opposition (“somewhat oppose” or “strongly oppose”; *n* = 51).

Considering playing status, responses indicated significant differences in support for temporary concussion substitutions [H _(6)_ = 33.352, *p* < 0.001], although the effect size was small (*ε*^2^ = 0.056). Here, support for temporary concussion substitutions was consistently high across all groups, with most responses falling within the “strongly favour” or “somewhat favour” categories (see Table 7). Opposition to temporary substitutions (with “somewhat oppose” or “strongly oppose” responses), was observed in a small proportion of respondents across several groups, including retired players (5.9%), Premier League (16.7%), Championship (12.5%), League One (16.1%), and League Two (15.8%) players. No opposition responses were recorded among players competing in the women's leagues. *Post-hoc* analyses revealed significant differences in support for temporary concussion substitutions between retired players and League One players (*p* = 0.005) and between retired players and League Two players (*p* < 0.001).

**Table 7:** The impact of playing status on support for temporary concussion substitutions.

Playing Status	Response Option	Number of Responses	Proportion of Sample (%)
Premier League	Strongly Favour	5	83.3
	Somewhat Favour	-	-
	Neutral	-	-
	Somewhat Oppose	-	-
	Strongly Oppose	1	16.7
Championship	Strongly Favour	23	57.5
	Somewhat Favour	5	12.5
	Neutral	7	17.5
	Somewhat Oppose	2	5.0
	Strongly Oppose	3	7.5
League One	Strongly Favour	11	35.5
	Somewhat Favour	5	16.1
	Neutral	10	32.3
	Somewhat Oppose	4	12.9
	Strongly Oppose	1	3.2
League Two	Strongly Favour	43	42.6
	Somewhat Favour	22	21.8
	Neutral	20	19.8
	Somewhat Oppose	9	8.9
	Strongly Oppose	7	6.9
Women's Super League	Strongly Favour	7	46.7
	Somewhat Favour	4	26.7
	Neutral	4	26.7
	Somewhat Oppose	-	-
	Strongly Oppose	-	-
FA Women's Championship	Strongly Favour	3	100.0
	Somewhat Favour	-	-
	Neutral	-	-
	Somewhat Oppose	-	-
	Strongly Oppose	-	-
Retired	Strongly Favour	260	64.4
	Somewhat Favour	80	19.8
	Neutral	40	9.9
	Somewhat Oppose	9	2.2
	Strongly Oppose	15	3.7

Further, support for temporary concussion substitutions differed by years of professional playing experience [H _(3)_ = 14.501, *p* = 0.002], with a small effect size (*ε*^2^ = 0.024). Specifically, the proportion of respondents selecting “strongly favour” increased with playing experience, rising from 40.6% among those who had played full-time for 1–3 years to 64.1% among those who had played for 11 years or more (see Table 8). *Post-hoc* analyses indicated a significant difference in support for temporary concussion substitutions between those with 11 or more years of professional playing experience and those with from between 1 and 3 years of professional playing experience (*p* = 0.005) only.

**Table 8:** The impact of years of professional playing experience on support for temporary concussion substitutions.

Years Played	Response Option	Number of Responses	Proportion of Sample (%)
1–3 Years	Strongly Favour	28	40.6
	Somewhat Favour	20	29.0
	Neutral	17	24.6
	Somewhat Oppose	1	1.4
	Strongly Oppose	3	4.3
4–6 Years	Strongly Favour	55	54.5
	Somewhat Favour	14	13.9
	Neutral	17	16.8
	Somewhat Oppose	7	6.9
	Strongly Oppose	8	7.9
7–10 Years	Strongly Favour	51	56.7
	Somewhat Favour	19	21.1
	Neutral	11	12.2
	Somewhat Oppose	4	4.4
	Strongly Oppose	5	5.6
11 + years	Strongly Favour	218	64.1
	Somewhat Favour	63	18.5
	Neutral	36	10.6
	Somewhat Oppose	12	3.5
	Strongly Oppose	11	3.2

### Perceptions of heading and long-term brain health risk

Responses indicated significant differences related to concerns about heading the ball across playing status [H _(6)_ = 28.348, *p* < 0.001], although the effect size was small (*ε*^2^ = 0.047). Specifically, among retired players, 28.7% indicated they were “extremely concerned” about heading-related risks. In contrast, the most frequent response among Premier League players (50%) was “moderately concerned”, most Championship (35%) and League One players (32.3%) were “not at all concerned”, while most League Two (29.7%) and Women's Super League players (26.7%) were “slightly concerned” about heading-related risks. *Post-hoc* analyses revealed a significant difference in concerns about heading the ball between retired players and League Two players (*p* = 0.001) only.

Similarly, concerns about long-term brain health and CTE risk differed significantly across playing status [H _(6)_ = 19.995, *p* = 0.003], with a small effect size (*ε*^2^ = 0.033). The combined responses of “extremely concerned” or “moderately concerned” were the most common among retired players (55.4%), Championship players (37.5%), League One players (35.5%), League Two players (42.6%), Women's Super League players (46.7%), and FA Women's Championship players (66.7%). The only exception was among Premier League respondents, where the most frequent response was “somewhat concerned” (66.7%). Notably, 30% of Championship respondents indicated that they were “not at all concerned” about their long-term brain health and CTE risk (see Table 9). *Post-hoc* analyses indicated a significant difference in concerns about long-term brain health and CTE risk between retired players and Championship players (*p* = 0.029) only.

**Table 9:** The impact of playing status on concerns about heading the ball and long-term brain health and CTE risk.

Playing Status	Response Option	Heading Risks		CTE Risks	
		Number of Responses	Proportion of Sample (%)	Number of Responses	Proportion of Sample (%)
Premier League	Extremely Concerned	-	-	1	16.7
	Moderately Concerned	3	50.0	1	16.7
	Somewhat Concerned	2	33.3	4	66.7
	Slightly Concerned	1	16.7	-	-
	Not At All Concerned	-	-	-	-
Championship	Extremely Concerned	4	10.0	5	12.5
	Moderately Concerned	9	22.5	10	25.0
	Somewhat Concerned	9	22.5	9	22.5
	Slightly Concerned	4	10.0	4	10.0
	Not At All Concerned	14	35.0	12	30.0
League One	Extremely Concerned	3	9.7	5	16.1
	Moderately Concerned	4	12.9	6	19.4
	Somewhat Concerned	7	22.6	7	22.6
	Slightly Concerned	7	22.6	10	32.3
	Not At All Concerned	10	32.3	3	9.7
League Two	Extremely Concerned	15	14.9	28	27.7
	Moderately Concerned	17	16.8	15	14.9
	Somewhat Concerned	10	9.9	14	13.9
	Slightly Concerned	30	29.7	27	26.7
	Not At All Concerned	29	28.7	17	16.8
Women's Super League	Extremely Concerned	3	20.0	2	13.3
	Moderately Concerned	3	20.0	5	33.3
	Somewhat Concerned	2	13.3	1	6.7
	Slightly Concerned	4	26.7	5	33.3
	Not At All Concerned	3	20.0	2	13.3
FA Women's Championship	Extremely Concerned	1	33.3	1	33.3
	Moderately Concerned	1	33.3	1	33.3
	Somewhat Concerned	-	-	-	-
	Slightly Concerned	-	-	-	-
	Not At All Concerned	1	33.3	1	33.3
Retired	Extremely Concerned	116	28.7	143	35.4
	Moderately Concerned	81	20.0	81	20.0
	Somewhat Concerned	65	16.1	68	16.8
	Slightly Concerned	81	20.0	78	19.3
	Not At All Concerned	61	15.1	34	8.4

Further, years of professional playing experience indicated significant differences in concerns about heading related-risks [H _(3)_ = 27.757, *p* < 0.001, *ε*^2^ = 0.046] and long-term brain health and CTE risk [H _(3)_ = 44.818, *p* < 0.001, *ε*^2^ = 0.075]. In both instances, the proportion of respondents reporting that they were “extremely” or “moderately concerned” increased with playing experience, rising from 24.6% among those who had played full-time for 1–3 years to 49.4% among those who had played for 11 years or more for heading-related risks and from 30.4% among those who had played full-time for 1–3 years to 59.4% among those who had played for 11 years or more for long-term brain health and CTE risk (see Table 10). *Post-hoc* analyses for concerns about heading the ball revealed significant differences between those with 11 or more years of professional playing experience and those with from between 4 and 6 years of professional playing experience (*p* = 0.023) and between those with 11 or more years of professional playing experience and those with from between 1 and 3 years of professional playing experience (*p* < 0.001). Similarly, *post-hoc* analyses for long-term brain health and CTE risk indicated significant differences between those with 11 or more years of professional playing experience and those with from between 7 and 10 years of professional playing experience (*p* = 0.013), between those with 11 or more years of professional playing experience and those with from between 4 and 6 years of professional playing experience (*p* < 0.001), and between those with 11 or more years of professional playing experience and those with from between 1 and 3 years of professional playing experience (*p* < 0.001).

**Table 10:** The impact of years of professional playing experience on concerns about heading the ball and long-term brain health and CTE risk.

Years Played	Response Option	Heading Risks		CTE Risks	
		Number of Responses	Proportion of Sample (%)	Number of Responses	Proportion of Sample (%)
1–3 Years	Extremely Concerned	5	7.2	11	15.9
	Moderately Concerned	12	17.4	10	14.5
	Somewhat Concerned	9	13.0	8	11.6
	Slightly Concerned	21	30.4	19	27.5
	Not At All Concerned	22	31.9	21	30.4
4–6 Years	Extremely Concerned	17	16.8	22	21.8
	Moderately Concerned	21	20.8	19	18.8
	Somewhat Concerned	18	17.8	16	15.8
	Slightly Concerned	18	17.8	30	29.7
	Not At All Concerned	27	26.7	14	13.9
7–10 Years	Extremely Concerned	18	20.0	18	20.0
	Moderately Concerned	19	21.1	22	24.4
	Somewhat Concerned	12	13.3	21	23.3
	Slightly Concerned	23	25.6	18	20.0
	Not At All Concerned	18	20.0	11	12.2
11 + years	Extremely Concerned	102	30.0	134	39.4
	Moderately Concerned	66	19.4	68	20.0
	Somewhat Concerned	56	16.5	58	17.1
	Slightly Concerned	65	19.1	57	16.8
	Not At All Concerned	51	15.0	23	6.8

### Concussion education

When asked whether they had received education regarding concussion, slightly more respondents reported that they had not received concussion education compared with those who had (*n* = 326 “no”, *n* = 274 “yes”).

There was no significant difference in support for temporary concussion substitutions between those who had and had not received concussion education (Z = −1.823, *p* = 0.068), with a negligible effect size (*r* = 0.074) (see Table 11).

**Table 11:** The impact of concussion education on support for temporary concussion substitutions.

Concussion Education	Response Option	Number of Responses	Proportion of Sample (%)
Yes	Strongly Favour	174	63.5
	Somewhat Favour	43	15.7
	Neutral	34	12.4
	Somewhat Oppose	10	3.6
	Strongly Oppose	13	4.7
No	Strongly Favour	178	54.6
	Somewhat Favour	73	22.4
	Neutral	47	14.4
	Somewhat Oppose	14	4.3
	Strongly Oppose	14	4.3

However, respondents who reported receiving concussion education demonstrated significantly greater concern regarding the risks associated with heading the ball (Z = −2.266, *p* = 0.023, *r* = 0.093) and potential long-term brain health and CTE risk (Z = −3.734, *p* < 0.001, *r* = 0.152) compared with those who said they had not received concussion education (see Table 12).

**Table 12:** The impact of concussion education on concerns about heading-related risks and long-term brain health and CTE risk.

Concussion Education	Response Option	Heading Risks		CTE Risks	
		Number of Responses	Proportion of Sample (%)	Number of Responses	Proportion of Sample (%)
Yes	Extremely Concerned	73	26.6	98	35.8
	Moderately Concerned	57	20.8	62	22.6
	Somewhat Concerned	42	15.3	45	16.4
	Slightly Concerned	59	21.5	49	17.9
	Not At All Concerned	43	15.7	20	7.3
No	Extremely Concerned	69	21.2	87	26.7
	Moderately Concerned	61	18.7	57	17.5
	Somewhat Concerned	53	16.3	58	17.8
	Slightly Concerned	68	20.9	75	23.0
	Not At All Concerned	75	23.0	49	15.0

## Discussion

The present study examined current and former professional football players’ perceptions of brain health, concussion management, and potential long-term neurological risks. Several important findings emerged. First, a greater proportion of participants reported experiencing a suspected concussion during their playing career than those who reported receiving a formal diagnosis, suggesting that concussions may occur more frequently than they are formally identified within professional football. Second, while many players indicated that they would prefer to remain on the field following a suspected concussion (if possible), the majority also reported that players themselves should not (ultimately) be responsible for making decisions about whether to continue playing. Third, there was strong support among respondents for the introduction of temporary concussion substitutions in professional football, particularly among retired players and those with longer playing careers. Fourth, many respondents expressed concern about the potential risks associated with heading the ball and the possible development of long-term neurological conditions such as CTE. Finally, while many players reported having received concussion education, a substantial proportion had not, highlighting gaps in educational provision within the professional game. Taken collectively, these findings provide insight into how professional football players perceive concussion management and brain health risks, as well as highlighting several areas in which policy, education, and medical practice within the sport may be strengthened.

### Concussion experience

Results from this study highlighted a discrepancy between the number of players who reported receiving a formal diagnosis of concussion and those who believed they had experienced a concussion while playing. Such findings suggest that concussions can go unrecognised or undiagnosed within professional football (19). Similar discrepancies between suspected and diagnosed concussions have been reported in other sporting populations, where athletes frequently report experiencing symptoms consistent with concussion without receiving medical confirmation (5). While international consensus statements emphasise that effective concussion management depends on early recognition and immediate removal from play (1), concussion diagnosis in sport often relies (at least in part) on athletes recognising and reporting symptoms to medical staff. Yet, many players fail to report symptoms of concussion due to uncertainty about injury severity, delayed symptom onset, or perceived pressure to continue playing (20). As such, findings from the present study reinforce the idea that reliance on athlete self-reporting may limit the effectiveness of concussion detection in competitive environments. Although many respondents indicated that they would prefer to remain on the field following a suspected concussion, most players also suggested that athletes should not be responsible for determining whether it is safe to continue playing. Athletes often experience conflicting motivations when evaluating injuries, balancing personal health considerations with perceived expectations from teammates, coaches and more (20). Thus, the findings presented herein highlight the importance of systems that support independent medical decision-making in concussion management. These findings also provide context for the strong player support observed in this study for the proposed introduction of temporary concussion substitutions in professional football.

### Support for temporary concussion substitutions

Results from this study identified strong support among respondents for the introduction of temporary concussion substitutions in professional football. These findings are particularly relevant in the context of ongoing discussions among football governing bodies such as FIFA, UEFA, and the International Football Association Board regarding potential modifications to concussion management protocols in the sport.

Currently, on-pitch concussion assessments in football are conducted under time constraints (typically <3 min), and removing a player from the field for medical evaluation can place the team at a temporary competitive disadvantage. In contrast, sports such as rugby union allow temporary substitutions for players to undergo head injury assessments without disadvantaging their team (9). These systems were introduced specifically to support more accurate concussion detection and to prioritise athlete welfare (8). The strong support for temporary concussion substitutions observed in this study suggests that professional football players recognise the potential benefits of such measures. Indeed, temporary substitutions may facilitate more comprehensive medical evaluation following a suspected concussion, reduce the likelihood that concussed players remain on the field, and help ensure that decisions regarding removal from play are guided by medical professionals rather than athletes themselves (8). By reducing concerns about disadvantaging the team, such measures may also reduce perceived pressure on players to continue playing following a head injury (21, 22). Higher levels of support among retired players and those with longer playing careers may also reflect greater awareness of the potential long-term health consequences of repeated head impacts. Further, retired players may have greater ‘temporal distance’ from the competitive pressures experienced during active playing careers, which may influence their perspectives on player safety measures.

### Perceptions of heading and long-term brain health risk

A substantial proportion of respondents expressed concern regarding both the potential risks associated with heading the ball and the possibility of developing long-term neurological conditions such as CTE. These concerns were particularly evident among retired players and among those with longer playing careers. Such findings are also consistent with increasing public and scientific attention surrounding the possible long-term neurological consequences of repeated head impacts in football (23). Here, epidemiological research involving former professional football players has reported higher rates of neurodegenerative disease compared with the general population (12). In addition, neuropathological studies in athletes exposed to repetitive head trauma have identified CTE associated with repeated head impacts (14). With regards to the differences observed between current and retired players, these findings might be explained by the ‘proximity of risk’. Specifically, current players may be more focused on immediate performance demands and career progression, while retired players may be more likely to reflect on potential long-term health consequences following the end of their careers. Regardless, these findings highlight the importance of continued research and policy development to mitigate potential risks associated with repeated head impacts. Thus, governing bodies and player organisations may wish to consider strategies that support brain health across the playing career and into retirement, including reducing exposure to repetitive head impacts and ensuring appropriate long-term medical support for former players^1^.

### Concussion education

The findings from this study highlighted potential considerations regarding concussion education within professional football. Although many respondents reported having received concussion education, a substantial proportion indicated that they had not participated in such programmes. Given the important role that athletes play in recognising and reporting concussion symptoms (24), this finding suggests that more resources need to be made available to support concussion education efforts across the professional game. Previous research has demonstrated that concussion education programmes can improve athletes’ knowledge and attitudes regarding concussion symptoms and management (17). In the present study, players who reported receiving concussion education also demonstrated greater concern regarding the potential risks associated with heading the ball and the development of CTE. This suggests that education might contribute to increased awareness of potential brain health risks. Education programmes may also influence reporting behaviour (21). Indeed, athletes with greater knowledge of concussion symptoms may be more likely to recognise and report potential injuries to medical staff. However, previous research [e.g., (16)] has shown that increased knowledge does not always translate into behavioural change within competitive sporting environments. Therefore, education should be considered one component of a broader strategy to improve concussion management, alongside policy interventions and medical assessments designed to reduce pressures on athletes to continue playing following head injury. Increasing access to concussion education programmes across professional football, including the introduction of regular refresher training, may help to ensure that players remain informed about evolving scientific evidence regarding concussion and long-term brain health.

However, these findings should be interpreted within the specific context of professional football. Here, it should be noted that systematic concussion education within the professional game was only formally implemented in 2023, after the present survey was launched. As a result, concussion education prior to this period is likely to have been variable in both availability and content. While many respondents reported receiving concussion education, it is not possible to determine what information was covered, who delivered it, or the quality and consistency of such provision. This is particularly relevant when interpreting player awareness of CTE, as concerns regarding long-term neurological risk may not arise solely from formal education. Indeed, players may also derive knowledge from wider media coverage, public discussion of former players diagnosed with neurodegenerative conditions, or experiences involving former teammates and contemporaries. Thus, future research should examine the content, quality, and consistency of concussion education delivered within professional football, particularly among active players who are now more likely to receive structured provision, including information about CTE within concussion education delivered by the PFA. Longitudinal research may also help to identify changing trends in player knowledge over time. In addition, future studies should explore players’ specific beliefs regarding the relationship between concussion exposure and CTE, including whether athletes perceive concussions as a direct cause of later neurological disease. Such work would help inform more targeted and evidence-based educational approaches across the professional game.

### Strengths and limitations

A strength of the present study was the inclusion of both current and former professional football players across multiple playing levels, providing insight into how perceptions of brain health may vary across different stages of a playing career. This study also captures professional athlete perspectives on concussion management and brain health policy, which remain relatively underrepresented within the sport medicine literature (15). As athletes are directly affected by concussion protocols and safety regulations, their views represent an important but often overlooked component of policy development and player welfare discussions. Yet, the findings presented herein demonstrate that players themselves recognise several challenges associated with concussion reporting and management, including the difficulty of making return-to-play decisions during competition. Therefore, incorporating athlete perspectives into policy discussions may provide valuable insight into the practical realities of concussion management in professional sport. Indeed, athlete-centred approaches to policy development may help ensure that player welfare initiatives are both effective and aligned with the lived experiences of professional athletes.

Several limitations associated with this study should also be acknowledged. First, the cross-sectional survey design captures perceptions at a single point in time and does not allow causal relationships to be established. Second, the study relied on self-report data, which may be subject to recall bias when participants report past concussion experiences. Third, while the sample included players from multiple playing levels, the number of female respondents was relatively small, limiting the ability to conduct sex-specific analyses. Given that women's football has different concussion incidence rates and mechanisms compared to the men's game, findings from this research should be carefully considered in the context of professional women's football. Fourth, retired players – who made up a large proportion of the sample – did not report their highest level of participation, meaning respondents were treated as a single, homogenous group regardless of the league they retired from. Participation in the survey was also voluntary, which introduces the possibility of self-selection bias, whereby players with stronger views on brain health may have been more likely to participate. In addition, because the survey was conducted with members of the PFA, findings may reflect the organisational and cultural context of professional football in England and may not necessarily generalise to players in other national leagues. Thus, future research should consider combining survey-based approaches with qualitative methods, such as interviews or focus groups, to explore in greater depth the factors that shape players’ perceptions of concussion and brain health. Longitudinal research could also help examine how player attitudes evolve over time as concussion policies, medical protocols, and scientific knowledge continue to develop.

## Conclusion

This study examined current and former professional football players’ perceptions of brain health, concussion management, and long-term neurological risk. Findings suggest that suspected concussions may occur more frequently than formally diagnosed injuries, highlighting the importance of improving concussion recognition and assessment procedures within professional football. Players also expressed strong support for the introduction of temporary concussion substitutions, which may facilitate more comprehensive medical evaluation and reduce pressure on athletes to remain on the field following a head injury. Many respondents also expressed concern regarding potential long-term neurological risks associated with repeated head impacts, particularly among retired players and those with longer playing careers. These findings highlight the importance of concussion education, medical assessment protocols, and the incorporation of athlete perspectives into the development of player welfare policies. By directly surveying the views of professional footballers, this study contributes novel evidence to ongoing discussions regarding concussion management, brain health, and the protection of long-term player wellbeing in the sport.

## Data Availability

The raw data supporting the conclusions of this article will be made available by the authors, without undue reservation.

## Footnotes

^1^
White AJ, Howarth NE, Nowkinski CJ, Batten J, Alosco ML, Buckland ME, et al. CTE Prevention protocol in practice: the drive to improve the brain health of professional footballers. *Front Neurol.* (2026). [Under Review].
